# Dependence of Liquid Supercooling on Liquid Overheating Levels of Al Small Particles

**DOI:** 10.3390/ma9010007

**Published:** 2015-12-24

**Authors:** Qingsong Mei, Juying Li

**Affiliations:** 1 Department of Materials Engineering, School of Power and Mechanical Engineering, Wuhan University, Wuhan 430072, China; 2 School of Mechanical Engineering, Wuhan Polytechnic University, Wuhan 430023, China; jyli@whpu.edu.cn

**Keywords:** liquid overheating, liquid supercooling, solidification, thermal history, Al particles

## Abstract

The liquid thermal history effect on liquid supercooling behavior has been found in various metals and alloys; typically the degree of liquid supercooling (Δ*T^−^*) increases with the increase of liquid overheating (Δ*T^+^*) up to several to tens of degrees above the equilibrium melting point (*T*_0_). Here we report quantitative experimental measurements on the Δ*T^−^*-Δ*T^+^* relationship of Al small particles encapsulated in Al_2_O_3_ shells by using a differential scanning calorimeter. We find a remarkable dependence of Δ*T^−^* on Δ*T^+^* of Al small particles, extending to at least 340 °C above *T*_0_ of Al (~1.36*T*_0_), which indicates the existence of temperature-dependent crystallization centers in liquid Al up to very high liquid overheating levels. Our results demonstrate quantitatively the significant effect of liquid thermal history on the supercooling behavior of Al and its alloys, and raise new considerations about the dependence of Δ*T^−^* on Δ*T^+^* at very high Δ*T^+^* levels.

## 1. Introduction

Solidification and melting are transformations basic to technological applications such as casting, crystal growth, and glass formation. Historically, the liquid thermal history was discovered to have a significant effect on the liquid supercooling behavior and thus, the nucleation and growth of crystals and their qualities [1,2,3,4,5,6,7,8,9,10,11,12,13]. The thermal history effect can be quantified by the relationship between liquid overheating (Δ*T^+^*), which is measured by the difference between liquid temperature and the equilibrium melting point (*T*_0_), and liquid supercooling (Δ*T^−^*), which is measured by the difference between *T*_0_ and the temperature of solidification. Thus far, the dependence of Δ*T^−^* on Δ*T^+^* has been investigated in metals/semimetals (Bi, Sn, Ga) [2,3,4,5,11,14] and alloys [6,7,8,9,10,11]; typically Δ*T^−^* increases with an increase of Δ*T^+^* up to several to tens of degrees above *T*_0_. Despite the fundamental and technical importance, the underlying mechanism of this phenomenon in relation to the liquid structure and the kinetics of heterogeneous nucleation of solidification is not well understood. Turnbull [14] suggested that crystals in microcavities, which are on the container or surface of impurity particles inside a melt, can be retained above *T*_0_, *i.e.*, they have elevated melting points. These retained crystals can serve as nuclei for solidification at a certain supercooling level, which results in an increase of Δ*T^−^* with the increase of Δ*T^+^* [14].

The cavity theory has been quantitatively validated by various experimental results [2,3,4,5], but it was considered to be not general, e.g., the dependence of Δ*T^−^* on Δ*T^+^* in Bi, Sn, SnSb and SnPb was found to be either continuous or discontinuous [10,11], which points to the evolution of transient short range order structures in the liquid state. Obviously, more investigations of the Δ*T^+^*-Δ*T^−^* relationship (thermal history effect) in metals and alloys are needed. In the casting of Al alloys, liquid thermal history effects on the microstructure of solidification have been reported [15,16]. Hereby, a quantitative study of the dependence of Δ*T^−^* on Δ*T^+^* in the simple metallic system Al is considered to be interesting. For experimental measurements of the Δ*T^+^*-Δ*T^−^* relationship, a precise control of temperature and heating/cooling rate is needed, and extraneous effects (e.g., oxidation) should be excluded [3,4]. Meanwhile, in bulk continuous systems, the factors affecting the solidification process are rather accidental, and the crystallization centers in one region may have an effect on the freezing behavior of the whole. In this study, we prepared samples of Al particles encapsulated in Al_2_O_3_ shells to eliminate possible effects of oxidation and coalescence at high temperatures. By using differential scanning calorimeter (DSC), we quantitatively measured the Δ*T^+^*-Δ*T^−^* relationship of encapsulated Al particles and found a remarkable dependence of Δ*T^−^* on Δ*T^+^* that extends to ultrahigh Δ*T^+^* levels.

## 2. Materials and Methods

Al small particles were prepared by means of active H_2_ plasma evaporation and condensation, using bulk Al (with a purity of 99.8%) as the starting material. The particles were *in situ* passivated at room temperature before exposure to air. The passivated Al particles were further oxidized in air to produce a thick surface oxide shell. Samples with different oxidation extents were produced by changing the temperature/duration of oxidation.

The morphology and size of Al particles was investigated by a transmission electron microscope (TEM, Philips, Amsterdam, The Netherlands) and a high-resolution transmission electron microscope (HRTEM, JEOL, Tokyo, Japan), which was conducted on a Philips EM 420 microscope (Philips) with an accelerating voltage of 100 kV and a JEM 2010 high-resolution microscope (JEOL) with an accelerating voltage of 200 kV, respectively. Samples for TEM and HRTEM observations were prepared by dispersing the particles in ethanol by sonication and then, dropping on a carbon-coated copper grid. Formation of an oxide shell on the Al particle surface was confirmed by TEM and HRTEM observations. The surface oxide shell was found to be able to retain the shape of Al particles effectively upon heating and cooling so that the particle can melt and freeze independently.

Thermal analysis was performed on a Netzsch high-temperature differential scanning calorimeter (DSC 404C, Netzsch, Selb, Germany). The sample in Al_2_O_3_ crucible was measured in a dynamic Ar atmosphere with a gas flow rate of 50 mL/min and a heating/cooling rate of 20 °C/min. The temperature and enthalpy were calibrated by the melting endotherms of pure In, Al and Ag.

## 3. Results and Discussion

**Figure 1:**
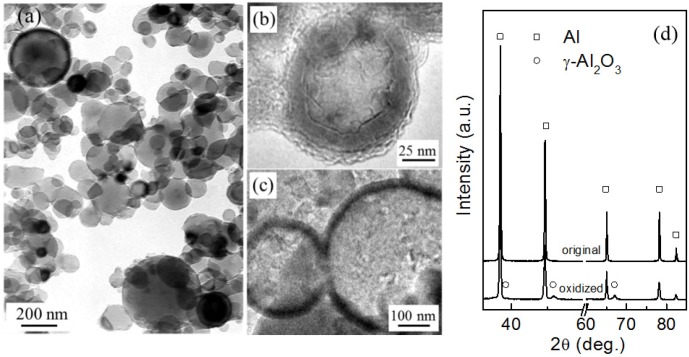
(**a**) A bright-field transmission electron microscope (TEM) image of the as-evaporated (original) Al particles; (**b**,**c**) high-resolution transmission electron microscope (HRTEM) images of small and large Al particles oxidized at 500 °C for 90 min, showing existence of the surface oxide shell; (**d**) XRD profiles of the original and oxidized (at 500 °C for 90 min) Al particles.

As shown in Figure 1a, the original Al particles are nearly spherical in shape with sizes mostly ranged from ~30 to ~500 nm. Al particles were encapsulated in Al_2_O_3_ shells after thermal oxidation at 500 °C for 90 min in air (Figure 1b,c). X-ray diffraction (XRD) analysis also confirmed the formation of oxide shells (Figure 1d). The Al_2_O_3_ shells are able to prevent the coalescence of Al nanoparticles upon heating and retain the particles shape even at temperatures well above *T*_0_ [17,18], allowing for the independent melting and solidification of encapsulated Al particles.

**Figure 2:**
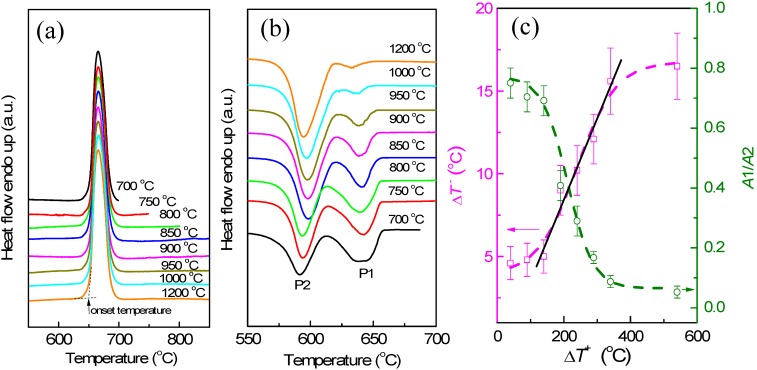
(**a**) Differential scanning calorimeter (DSC) melting curves of the encapsulated Al particles (oxidized at 500 °C for 90 min) heated to different temperatures as indicated, showing the good repeatability of melting endotherms after several thermal cycles. The definition of the onset temperature of DSC peak is indicated in (a); (**b**) DSC curves of the encapsulated Al particles (oxidized at 500 °C for 90 min) cooled from different temperatures as indicated, showing variations of the two freezing exotherms P1 and P2 with liquid temperature; (**c**) Variations of Δ*T^−^* of P1 and relative area ratio of P1 to P2 (*A*1/*A*2) as functions of Δ*T^+^*. In (c), the dashed lines are guides for eyes, and the solid line is a linear fitting of the data in the Δ*T^+^* range of 140–340 °C.

**Figure 3:**
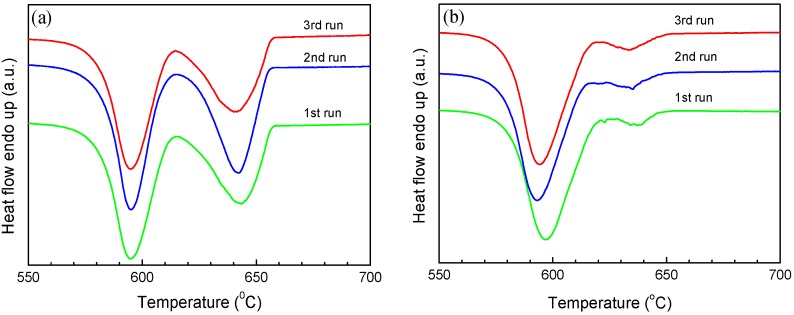
DSC cooling curves of the encapsulated Al particles (oxidized at 500 °C for 90 min) for different liquid overheating temperatures (**a**) 800 °C and (**b**) 1000 °C. Each run was performed immediately after a previous 1200 °C heating-cooling cycle.

**Figure 4:**
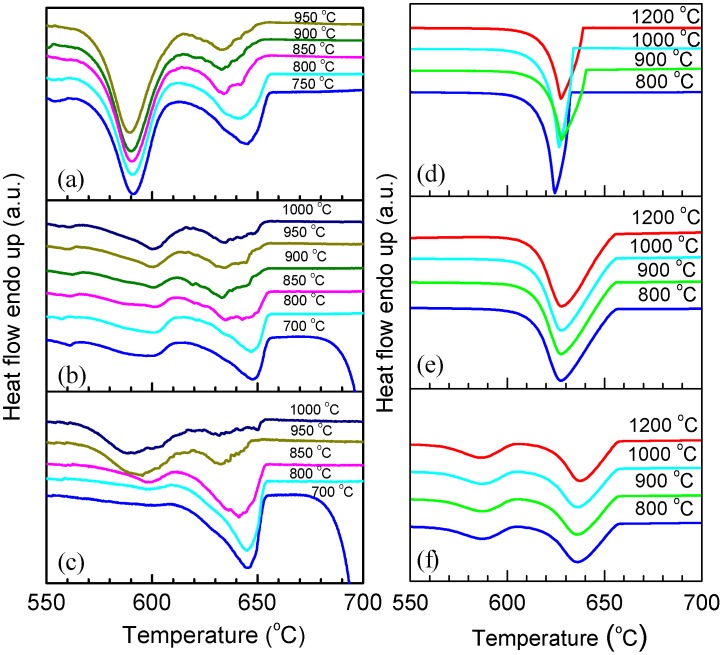
DSC curves corresponding to the cooling from different temperatures as indicated for different samples. (**a**) Al particles oxidized at 700 °C for 90 min; (**b**) Al particles oxidized at 800 °C for 180 min; (**c**) Al particles oxidized at 900 °C for 180 min; (**d**) 99.999% bulk Al; (**e**) 99.8% bulk Al; (**f**) Consolidated samples of oxidized Al particles (at 500 °C for 90 min) made by cold pressing.

The melting and solidification behaviors of encapsulated Al particles were measured by DSC. As shown in Figure 2a, melting endotherms of encapsulated Al particles are quite similar after several heating/cooling cycles, showing a reduced melting temperature (onset temperature of the melting peak: ~650 °C) due to the size-dependent melting point depression [19]. It is noted that previous studies [20,21,22,23] indicated the important effects of stress relaxation and damage of the oxide shell on the melting of Al nanoparticles coated by oxide shells, while in the present study the good repeatability of melting endotherms suggests the good thermal stability of encapsulated Al particles after several DSC thermal cycles, which may be attributed to the thicker oxide shells that were formed by thermal oxidation in the present study. Figure 2b demonstrates DSC curves of the Al particles cooled from different liquid temperatures/overheating levels. All DSC curves consist of two main exothermic peaks, one with small supercooling (P1) and another with larger supercooling (P2). Most importantly, the solidification behavior shows an evident dependence on the liquid temperature. For a quantitative illustration, we take Δ*T^−^* as the difference between the onset temperature of P1, and *T*_0_ of Al and Δ*T^+^*, as that between the liquid temperature and *T*_0_. As shown in Figure 2c, an evident dependence of Δ*T^−^* on Δ*T^+^* is identified: Δ*T^−^* increases with the increasing of Δ*T^+^*, most evidently in the Δ*T^+^* range of 140–340 °C. In Figure 2c, the relative area ratio of the two peaks (*A*1/*A*2) also shows a strong dependence on Δ*T^+^*, indicating that when cooled from higher liquid temperatures, more Al particles solidify at larger supercoolings. In our experiments, the holding time at which the liquid was kept at Δ*T^+^* (up to 1 h) was found to have little effect on the Δ*T^+^*-Δ*T^−^* relationship, and the supercooling behavior was only related to the highest temperature to which the liquid was heated, regardless of the intermediate temperatures at which it was held before solidification, in agreement with predictions by the cavity theory [14]. Compared to previous experiments using fast scanning calorimetry with a ultrahigh cooling rate up to the order of 10^5^ °C/min [2,14,24,25], the cooling rate in the present study is very small (20 °C/min), which can exclude the cooling rate effect on the solidification of Al particles cooled from different liquid temperatures and rationalize the independence of the holding time at Δ*T^+^*. To confirm the reproducibility of the results and clarify possible effects of the morphologies changes on the oxidized Al particles induced by the high-temperature annealing/heating on the freezing behavior, we carried out several heating-cooling cycles for different heating temperatures of the liquid and obtained DSC curves immediately after the 1200 °C heating-cooling cycle. As shown in Figure 3, Δ*T^−^* still shows an evident dependence on Δ*T^+^* regardless of the previous 1200 °C heating-cooling cycle, indicating that the freezing behavior is only dependent on the maximum liquid heating temperature before solidification. We also carried out DSC measurements on samples with different oxidation degrees prepared by changing oxidation temperature/duration, all showing a similar Δ*T^+^*-Δ*T^−^* relationship, *i.e.*, Δ*T^−^* increases with increasing Δ*T^+^* (Figure 4a–c). To our knowledge, such Δ*T^+^*-Δ*T^−^* relationship has not been reported in Al before. Moreover, the Δ*T^+^* level (up to 340 °C) to which the dependence of Δ*T^−^* on Δ*T^+^* extends in the present study is significantly larger than that observed in other metals and alloys (typically several to tens of degrees) [14].

For comparison, bulk Al samples with different impurity levels were also tested, as shown in Figure 4d–f. For bulk Al sample with a purity of 99.999%, the solidification behavior varies randomly for each heating-cooling cycle, which is due to the accidental nature of catalysts for nucleation of solidification. For bulk Al sample with a purity of 99.8%, the DSC cooling curves are similar for different Δ*T^+^*s, with a smaller supercooling than bulk Al sample with a higher purity level (99.999%), which simply can be attributed to the higher content of catalysts for nucleation. The supercooling behavior of the consolidated sample, made by cold-pressing the oxidized Al particles, remains almost unchanged for different Δ*T^+^*s although two exothermic peaks are also observed, *i.e.*, no dependence of Δ*T^−^* on Δ*T^+^* as that in the powder sample is observed. This is possible because, as mentioned above, in bulk continuous systems, crystallization centers in one region may affect the solidification behavior of the whole. It is also shown in Figure 4 that the solidification temperatures of P1 of the powder sample, the consolidated sample, and bulk Al sample with a purity of 99.8% are similar but different from that of the bulk Al sample with a purity of 99.999%, indicating the important effect of impurities.

**Figure 5:**
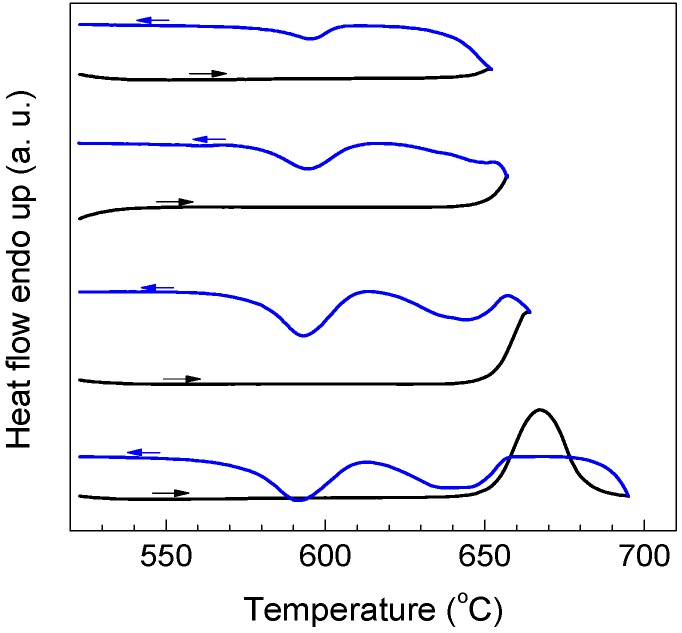
DSC partial melting-cooling curves for the encapsulated Al particles (oxidized at 500 °C for 90 min). Melting of the encapsulated Al particles was interrupted at different temperatures (as indicated) for 60 s before the cooling step.

For a particle/droplet, different nucleation mechanisms, namely bulk and surface nucleation, can have significant effects on the solidification behavior [24,25]. To identify the two solidification processes of Al particles, the sample was heated to different temperatures followed by cooling down so that only a part of Al particles in the sample were melted and solidified. As shown in Figure 5, Al particles melted at lower temperatures tend to solidify at larger supercoolings (P2), and those with higher melting temperatures at smaller supercoolings (P1). Since smaller particles have lower melting points than larger ones due to the size effect [19], it is believed that P1 corresponds to the solidification of larger Al particles while P2 corresponds to smaller ones. Previous studies [26,27] showed that the multi-stage solidification behaviors in various embedded particle/matrix systems can be related to the different catalyzing effects of impurities and matrix/coating. Hereby, we can further conclude that P1 corresponds to the solidification of larger particles (with more impurities) related to the catalyzing effect of impurities while P2 corresponds to smaller particles (with less impurities) catalyzed by the coating. It is noted that previous phase field simulations [28] indicated that Al melt can completely wet alumina with a contact angle of zero. However, interfacial energy between Al and Al_2_O_3_ is also dependent on factors such as crystallography, temperature, impurity, *etc*. Here, it is believed that upon solidification, heterogeneous nucleation on the Al/Al_2_O_3_ interface would be more likely than homogenous nucleation, as long as upon nucleation the Al/Al_2_O_3_ interface is formed so that the contact angle of solid Al on Al_2_O_3_ is between 0° and 180°, by e.g., forming a favored local crystallography. The relative change in peak areas shown in Figure 2c where the increasing Δ*T*^+^ is due to the modification of the two nucleation sites upon elevated temperature exposure: bulk heterogeneous nucleation (P1) and surface/interface heterogeneous nucleation (P2). The above analysis indicates that the dependence of Δ*T^−^* on Δ*T^+^* of encapsulated Al particles observed in the present experiment is related to effect of impurity (alloying) atoms despite the minute amount. Hereby, a critical problem is how the liquid overheating can lead to a transfer between the two nucleation mechanisms, *i.e.*, how bulk heterogeneous nucleation is suppressed by liquid overheating.

The dependence of Δ*T^−^* on Δ*T^+^* obtained in our study indicates the existence of temperature-dependent crystals in liquid Al up to a very high Δ*T^+^* (~340 °C ), unlike the transient short range orders as in the cases of semimetals and semiconductors [11,12]. Previous studies indicated that the crystals confined by a coating/matrix can be superheated metastably providing epitaxial interfaces are formed [19,29,30]. However, in our study, the Al-Al_2_O_3_ interface is non-epitaxial. Although a small pressure-induced superheating up to ~15 °C was observed [31], it cannot obviously account for the very large superheating (~340 °C) that is close to the inverse Kauzmann point (highest superheating limit) [32] in the present study. As mentioned above, the cavity theory [14] provided a possible explanation for the dependence of Δ*T^−^* on Δ*T^+^*. According to the cavity theory [14], a crystal contained in a cavity with a radius of *r* can remain unmelted at a certain liquid overheating level (Δ*T^+^*), given by:

(1) $$\Delta T^{+}=\frac{2T_{0}\sigma_{\text{sl}}\cos\theta }{\Delta H_{\text{m}}r}$$

where σ_sl_ is the solid-liquid interfacial energy of the host metal, θ is the contact angle between the cavity and the host liquid, and Δ*H*_m_ is the melting enthalpy of the host metal. The cavity theory predicted a linear relationship between Δ*T^+^* and Δ*T^−^* [14]:

(2) $$\Delta T^{-}/\Delta T^{+}=\tan\theta$$

In the present experiment, the Δ*T^+^*-Δ*T^−^* relationship in the Δ*T^+^* range of 140 to 340 °C is approximately linear, as shown in Figure 2. By linear fitting of the experimental data according to Equation (2), we get θ = 2.6°. Meanwhile, for $\Delta T_{\max}^{+}$= 340 °C and $\Delta T_{\min}^{+}$= 140 °C with available data of Δ*H*_m_ and σ_sl_ for Al [33], we get *r*_min_ = 0.6 nm and *r*_max_ = 1.5 nm from Equation (1), corresponding to the radius of the smallest and largest cavities, respectively. However, the existence of cavities with such extremely small sizes and contact angles is hardly reasonable, indicating the insufficiency of the cavity theory for interpreting the dependence of Δ*T^−^* on Δ*T^+^* extending to ultrahigh Δ*T^+^* in the present experiment. In a Ni-base superalloy, existence of Ni_3_(Al,Ti,Nb)-like clusters and residual MC (M = Nb, Ti) carbide in the alloy melt superheated to 1500 °C (170 °C above the liquidus temperature) was detected by high temperature X-ray diffraction, resulting in a liquid thermal history effect on solidification [6]. Hereby, a possible consideration is that the persisted crystals are not likely pure Al but alloyed clusters containing Al and other (impurity) atoms, as indicated by the impurity-related evidence of the dependence of Δ*T^−^* on Δ*T^+^* in the present study. However, for a better interpretation of the present results, direct experimental evidence for the size, surface energy, and melting point of these clusters is needed.

## 4. Conclusions

In summary, through quantitative experimental measurements, we found a remarkable dependence of Δ*T^−^* on Δ*T^+^* extending to ultrahigh Δ*T^+^* levels in Al small particles encapsulated in Al_2_O_3_ shells. While the analysis pointed to the existence of temperature-dependent crystallization centers in liquid Al at very high liquid temperatures, the cavity theory was found to be insufficient to interpret the present results. The present study highlighted quantitatively the significant effect of liquid overheating levels on the supercooling behavior of Al and Al alloys, and raised new considerations for the dependence of liquid supercooling on liquid overheating at very high liquid overheating levels.
